# High proportions of regulatory T cells in PBSC grafts predict improved survival after allogeneic haematopoietic SCT

**DOI:** 10.1038/bmt.2015.215

**Published:** 2015-09-21

**Authors:** R D Danby, W Zhang, P Medd, T J Littlewood, A Peniket, V Rocha, D J Roberts

**Affiliations:** 1 Department of Haematology Cancer and Haematology Centre, Oxford University Hospitals NHS Trust, Churchill Hospital, Oxford, UK; 2 NHS Blood and Transplant, John Radcliffe Hospital, Oxford, UK; 3 Radcliffe Department of Medicine, University of Oxford, Oxford, UK; 4 Department of Haematology, Derriford Hospital, Plymouth, UK

## Abstract

Regulatory T cells (Tregs) modulate immune responses and improve survival in murine transplant models. However, whether the Treg content of allogeneic cell grafts influences the outcome in human haematopoietic stem cell (HSC) transplantation is not well established. In a prospective study of 94 adult allogeneic PBSC transplants (60% unrelated; 85% reduced intensity conditioning), the median Treg (CD3^+^CD4^+^CD25^+^FOXP3^+^CD127^dim/−^) dose transplanted was 4.7 × 10^6^/kg, with Tregs accounting for a median of 2.96% of CD4^+^ T cells. Patients transplanted with grafts containing a Treg/CD4^+^ T-cell ratio above the median had a 3-year overall survival of 75%, compared with 49% in those receiving grafts with a Treg/CD4^+^ T-cell ratio below the median (*P*=0.02), with a 3-year non-relapse mortality of 13% and 35%, respectively (*P*=0.02). In multivariate analysis, a high graft Treg/CD4^+^ T-cell ratio was an independent predictor of lower non-relapse mortality (hazard ratio (HR), 0.30; *P*=0.02), improved overall survival (HR, 0.45; *P*=0.03) and improved sustained neutrophil (HR, 0.52; *P*=0.002), platelet (HR, 0.51; *P*<0.001) and lymphocyte (HR, 0.54; *P*=0.009) recovery. These data support the hypothesis that the proportion of Tregs in allogeneic HSC grafts influences clinical outcome and suggest that Treg therapies could improve allogeneic HSC transplantation.

## Introduction

Allogeneic haematopoietic stem cell transplantation (HSCT) is a curative therapy for many haematological disorders. However, despite continued improvements, HSCT is associated with significant morbidity and mortality.^1, 2^ In particular, the immunological disparity between donor and recipient can cause graft rejection or GvHD.^3^ T-cell depletion and systemic immunosuppressive therapy reduce the incidence and severity of these complications, but delay immune reconstitution and increase the risk of infection.^4, 5^ Furthermore, these strategies impair the GvL response, increasing the risk of relapse.^6^ Reducing the harmful immunological effects while sparing the GvL response could, therefore, improve HSCT outcomes.

Regulatory T cells (Tregs) have an important role in allogeneic HSCT. In animal models, cotransfer of CD4^+^CD25^+^ Tregs and CD4^+^CD25^−^ effector T cells into MHC-mismatched mice with leukaemia prevented lethal GvHD but allowed an effective GvL response.^7, 8^ In humans, reduced CD4^+^CD25^high^ cells, CD4^+^CD25^high^FOXP3^+^ cells and *FOXP3* mRNA in blood and tissues have been observed in patients with GvHD,^9, 10, 11, 12, 13^ whereas several strategies used to treat GvHD have been associated with *in vivo* Treg expansion.^14, 15, 16, 17^ Early phase clinical trials of adoptive transfer of *ex vivo* isolated and expanded Tregs have also been commenced.^18, 19, 20^ However, few studies have examined the influence of Tregs in HSC grafts with clinical outcomes. In this study, we hypothesised that higher proportions of Tregs (Tregs/CD4^+^ T cells) in PBSC grafts are associated with improved haematological, immunological and clinical outcomes following allogeneic HSCT.

## Materials and methods

### Patients

Ninety-four patients transplanted in Oxford (2009–2011) using allogeneic PBSC harvests were recruited (Table 1). Patients and donors were HLA-matched using high-resolution typing at HLA-A/B/C/DRB1/DQB1 (HLA-matched sibling (*n*=37), single HLA-Ag-mismatched sibling (*n*=1), 10/10 unrelated donor (*n*=39) and 9/10 unrelated donor (*n*=17)). GvHD prophylaxis was cyclosporin and methotrexate. Unrelated donor transplants also received alemtuzumab 60 mg (30 mg days −8/−7 (*n*=49)) or 90 mg (30 mg days −8/−7/−6 (*n*=7)). Alemtuzumab (60 mg) was also given in sibling transplants with a single HLA-mismatch (*n*=1) or Hodgkin's disease (*n*=1). Informed written consent was obtained in accordance with the Declaration of Helsinki. The study was approved by the Oxfordshire Research Ethics Committee (07/H0606/16) and Oxford University Hospitals NHS Trust.

### Flow cytometry

**Figure 1:**
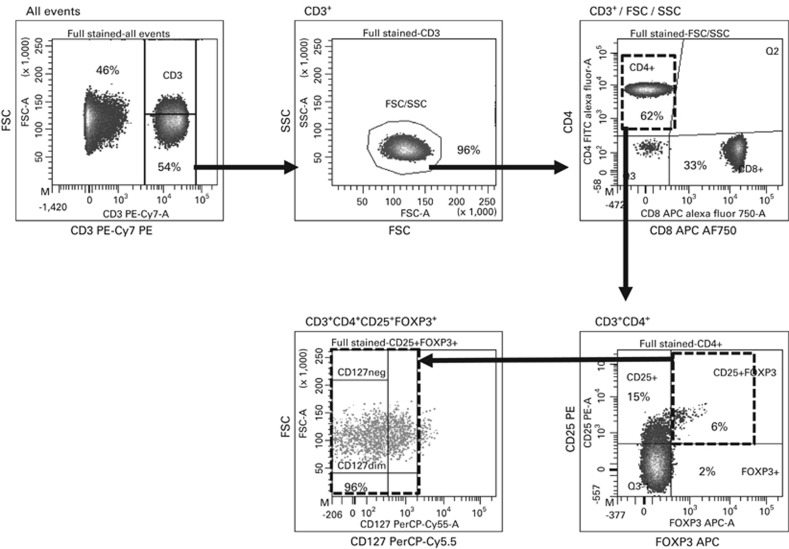
Flow cytometry for Tregs. PBMCs were stained with anti-CD3 PE-Cy7, -CD4 FITC, -CD8 APC-AF750, -CD25 PE, -FOXP3 APC, -CD127 PerCP-Cy5.5 and analysed on an LSRII Flow Cytometer (BD Biosciences, Oxford, UK). CD3^+^ cells were gated by CD3/FSC/SSC; CD3^+^CD4^+^CD8^−^ cells analysed for FOXP3 and CD25 expression; CD3^+^CD4^+^CD25^+^FOXP3^+^ cells analysed for expression of CD127. FSC, forward scatter; SSC, side scatter; Tregs, regulatory T cells.

Aliquots of G-CSF-stimulated PBSC grafts, with the total nucleated cell (TNC) and CD34^+^ dose, were obtained from the Stem Cell Laboratory (NHS Blood and Transplant, Oxford, UK). All samples were analysed fresh by flow cytometry (Figure 1 and Supplementary methods). Tregs (CD3^+^CD4^+^CD8^−^CD25^+^FOXP3^+^CD127^dim/−^) were expressed as the proportion of CD4^+^ T cells (Treg/CD4^+^ T-cell ratio) and dose (× 10^6^/kg).

### Outcomes

Neutrophil, platelet and lymphocyte recoveries were defined as the first of three consecutive days with a count >0.5, >50 and >1.0 × 10^9^/l, respectively, and recorded as sustained recovery in the absence of secondary graft failure. Acute GvHD (aGvHD) was graded using the Glucksberg criteria^21, 22^ and chronic GvHD (cGvHD) using the Seattle criteria.^23^ CMV activation was defined as detection of CMV DNA by PCR. Relapse was recorded as disease recurrence and non-relapse mortality (NRM) was defined as death without relapse. Overall survival was from the day of transplantation to death or last follow-up.

### Statistics

Categorical variables were tested using the *χ*^2^ test and continuous data using independent *t*-test or Mann–Whitney test. Correlations were analysed using Spearman's correlation. Backward linear regression was performed keeping variables with *P*<0.05. Overall survival was analysed using Kaplan–Meier. Cell recovery, GvHD, CMV, relapse and NRM were assessed using cumulative incidence with competing risk (relapse for NRM; death for other outcomes). Continuous variables were categorized using median or quartiles. Multivariate analysis was performed using the Cox proportional hazards (overall survival) or the Fine and Gray method (cumulative incidence). Variables with *P*<0.10 in univariate analysis were included and the model developed using a backward approach, keeping variables with *P*<0.05. As donor (sibling/unrelated) and alemtuzumab were strongly confounded, alemtuzumab was not included in analyses containing donor type. Statistical analysis was performed using SPSS 21 (IBM Corporation, New York, NY, USA) and R 3.0.2 (R Foundation for Statistical Computing, Vienna, Austria).

## Results

### Graft composition

The median TNC and CD34^+^ cell dose transplanted was 10.1 × 10^8^/kg (range, 2.7–37.6) and 6.3 × 10^6^/kg (range, 1.1–19.8), respectively (Table 1). Tregs accounted for a median of 2.96% (range, 0.81–8.56) CD4^+^ T cells, with a median dose of 4.7 × 10^6^/kg (range, 0.8–20.6). There were significant correlations between Treg dose and TNC, CD3^+^, CD19^+^ and CD3^−^CD56^+^ dose (*P*<0.0001) but not CD34^+^ cell dose (Supplementary Table S1). However, when Tregs were expressed as a proportion of CD4^+^ T cells, there were no significant correlations with either TNC, CD34^+^, CD3^+^, CD19^+^ or CD3^−^CD56^+^ cell dose.

PBSC grafts from sibling donors contained higher numbers of Tregs (4.35 vs 3.48 × 10^8^; *P*=0.01) and higher Treg/CD4^+^ T-cell ratios (0.033 vs 0.026; *P*=0.04). PBSC harvests requiring a second day of collection contained higher numbers of TNC (996 vs 753 × 10^8^; *P*<0.0001), CD3^+^ (360 vs 197 × 10^8^; *P*<0.0001) and Tregs (6.76 vs 3.51 × 10^8^; *P*<0.0001), but lower CD34^+^ cells (4.47 vs 5.39 × 10^8^; *P*=0.03). However, Treg/CD4^+^ T-cell ratios did not differ between 1- and 2-day harvests (*P*=0.74). Male donors showed an NS trend towards higher Treg/CD4^+^ T-cell ratios (*P*=0.07). There was no difference in Treg numbers or Treg/CD4^+^ T-cell ratios with donor age, ABO group or CMV serology. In multivariate analysis, the number of harvest days remained independently associated with Treg counts (*P*<0.001). In contrast, male donors (*P*=0.03) and sibling donors (*P*=0.01) were independently associated with higher Treg/CD4^+^ T-cell ratios.

To test the hypothesis that higher proportions of Tregs in the PBSC grafts are associated with improved outcomes following allogeneic HSCT, the study cohort was divided into two: above and below the median Treg/CD4^+^ T-cell ratio (Table 2). All multivariate analysis between Treg/CD4^+^ T-cell ratio and outcomes was adjusted for significant differences between these groups (donor age and donor type).

### Neutrophil, platelet and lymphocyte recovery

**Figure 2:**
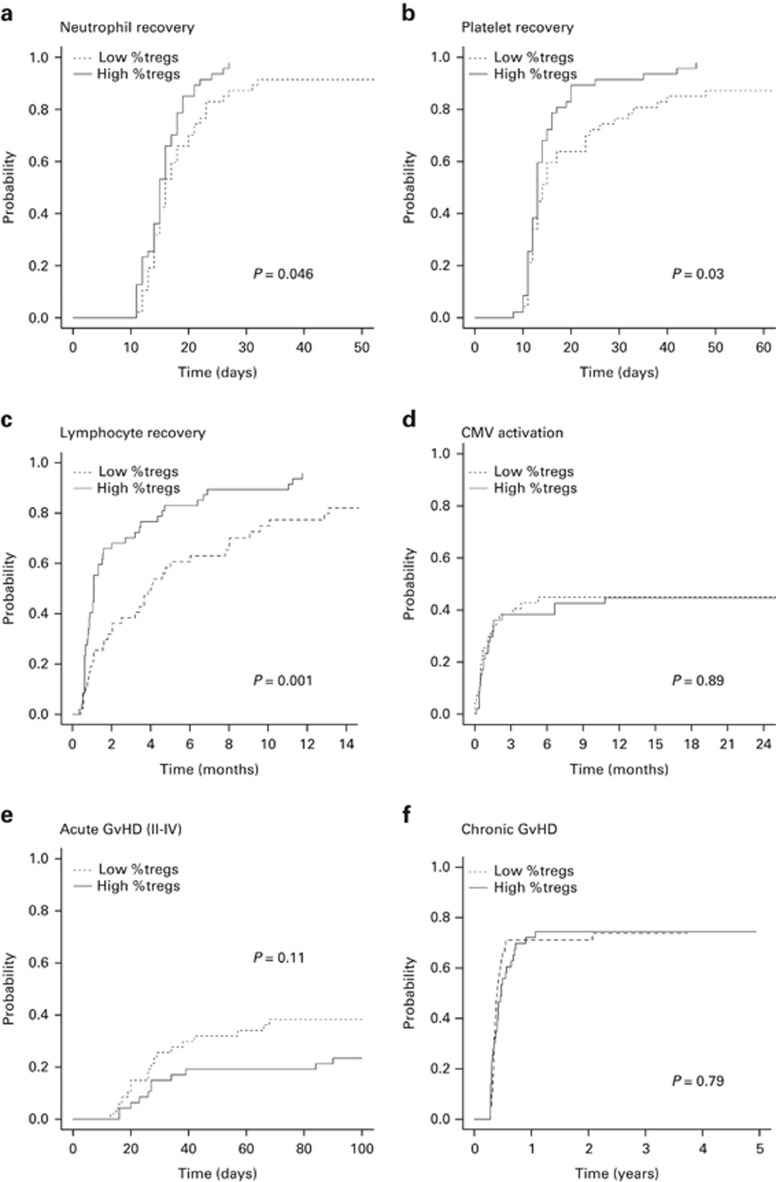
Haematopoietic recovery, CMV activation and GvHD. The cumulative incidence of (**a**) sustained neutrophil recovery (>0.5 × 10^9^/l), (**b**) sustained platelet recovery (>50 × 10^9^/l), (**c**) lymphocyte recovery (>1.0 × 10^9^/l), (**d**) CMV activation (CMV DNA detected by PCR), (**e**) acute GvHD (II–IV) and (**f**) chronic GvHD (all grades) according to the proportion of Tregs (Tregs/CD4^+^ T cells) in the graft. Low %Tregs, Tregs/CD4^+^ T cells below the median (dotted line); High %Tregs, Tregs/CD4^+^ T cells above the median (solid line); Tregs, regulatory T cells.

The cumulative incidence of neutrophil recovery (day 30) was 95% (95% confidence intervals (95% CI): 87–98), with a median of 16 days (range, 11–32). Two patients with initial recovery had secondary graft failure (days 25 and 27) and received a second allogeneic HSCT. Patients receiving higher proportions of Tregs in the graft had a higher cumulative incidence of sustained neutrophil recovery (98% (95% CI: 64–100) vs 87% (95% CI: 73–94); *P*=0.046) (Figure 2a and Table 3). This remained significant (hazard ratio (HR), 0.55 (95% CI: 0.37–0.81); *P*=0.003) when adjusting for significant differences between the groups. However, there was no significant difference in the incidence of neutrophil recovery when analysing Treg dose in the grafts (*P*=0.71). In multivariate analysis, Treg/CD4^+^ T-cell ratio (HR 0.52 (95% CI: 0.37–0.79); *P*=0.002) and CD34^+^ dose (HR, 0.65 (95% CI: 0.44–0.96); *P*=0.03) were independent predictors of neutrophil recovery (Table 4).

The cumulative incidence of platelet recovery (day 60) was 94% (95% CI: 86–97), with a median of 13 days (range, 8–137). Patients receiving higher proportions of graft Tregs had a higher cumulative incidence of sustained platelet recovery (98% (95% CI: 67–100) vs 87% (95% CI: 73–94); *P*=0.03), which remained significant after correcting for differences between the groups (HR, 0.54 (95% CI: 0.37–0.88); *P*=0.002) (Figure 2b). Treg dose was not associated with platelet recovery (*P*=0.95). In multivariate analysis, the Treg/CD4^+^ T-cell ratio (HR, 0.51 (95% CI: 0.34–0.76); *P*<0.001)), CD34^+^ cell dose (HR, 0.60 (95% CI: 0.40–0.88); *P*=0.01) and CD3^−^CD56^+^ dose (HR 1.52 (95% CI: 1.01–2.27); *P*=0.046) were independent predictors of platelet recovery (Table 4).

The cumulative incidence of lymphocyte recovery (6 months) was 72% (95% CI: 61–80), with a median of 99 days (range, 11–455). Patients receiving grafts containing higher than median Treg/CD4^+^ T-cell ratios had an increased incidence of lymphocyte recovery (83% (95% CI: 68–92)) compared with those with lower than Treg/CD4^+^ T-cell ratios (61% (95% CI: 45–73%)) (*P*=0.001) (Figure 2c). This was significant after adjusting for differences between the groups (HR, 0.56 (95% CI: 0.35–0.88); *P*=0.02). In multivariate analysis, higher Treg/CD4^+^ T-cell ratios (HR, 0.54 (95% CI: 0.34–0.86); *P*=0.009), sibling donors (HR, 0.30 (95% CI: 0.16–0.55); *P*<0.001) and female donors (HR, 0.59 (95% CI: 0.38–0.93); *P*=0.02) were independent predictors of improved lymphocyte recovery (Table 4).

### CMV activation

CMV activation postHSCT was observed in 42 patients at a median of 22 days (range, 0–327), with a cumulative incidence of 45% (95% CI: 35–55) at 1 year. Pretransplant positive CMV serology in the recipient (*P*<0.001) and donor (*P*<0.001), and the presence of an HLA-mismatch in the host-versus-graft (HvG) direction (*P*=0.01) were associated with the risk of CMV activation. However, neither the Treg/CD4^+^ T-cell ratio nor Treg dose in the graft were statistically significant (*P*=0.89 and *P*=0.15, respectively) (Figure 2d). In multivariate analysis, pretransplant CMV seropositive status of the recipient (HR, 21.5 (95% CI: 6.66–69.9); *P*<0.001) and donor (HR 2.50 (95% CI: 1.43–4.36); *P*=0.001) remained independently associated with CMV activation (Table 4).

### GvHD

The cumulative incidence grades II–IV and III–IV aGvHD was 31% (95% CI: 22–40) and 15% (95% CI: 9–23), respectively. The median onset for aGvHD (II–IV) was 27 days (range, 13–90). Single organ involvement was most frequent (61%) but skin, gut and liver were involved in 9% of cases. The incidence of aGvHD (II–IV) fell short of statistical significance when analysing either the Treg/CD4^+^ T-cell ratio (*P*=0.11) or Treg dose (*P*=0.11) in the graft (Figure 2e). In multivariate analysis, only disease status at transplant was a predictor of aGvHD (II–IV) (HR, 3.37 (95% CI: 1.48–7.66); *P*=0.004) (Table 4). The cumulative incidence of cGvHD was 74% (95% CI: 63–82) at 3 years, with an incidence of extensive cGvHD of 60% (95% CI: 48–70). Neither the Treg/CD4^+^ T-cell ratio (*P*=0.79) nor Treg dose (*P*=0.77) in the graft were associated with cGvHD (Figure 2f).

### Relapse and NRM

**Figure 3:**
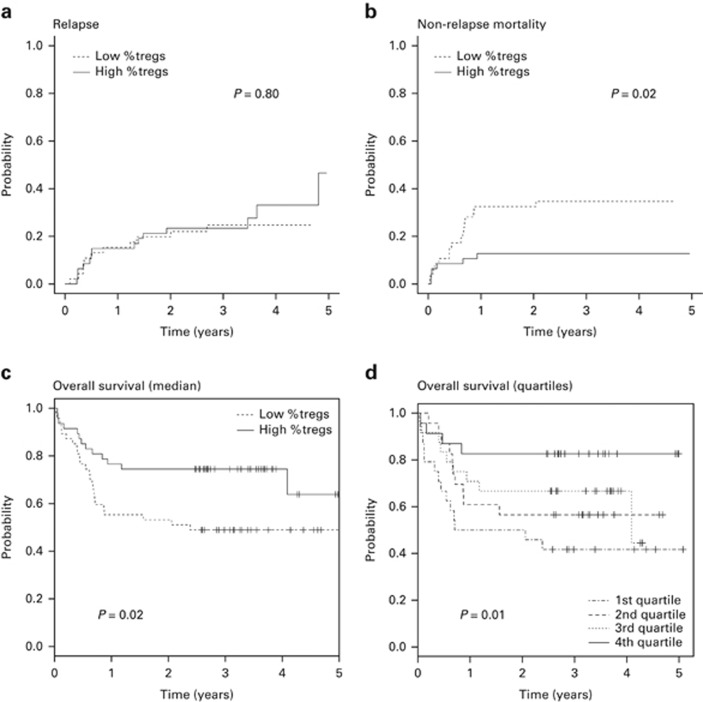
Relapse, NRM and overall survival. The cumulative incidence of (**a**) relapse and (**b**) NRM according to the proportion of Tregs (Tregs/CD4^+^ T cells) in the graft. (**c** and **d**) Overall survival according to the proportion of Tregs (Tregs/CD4^+^ T cells) in the graft. (**c**) Low %Tregs, Tregs/CD4^+^ T cells below the median (dotted line); High %Tregs, Tregs/CD4^+^ T cells above the median (solid line). (**d**) 1st Quartile, Tregs/CD4^+^ T cells <0.022; 2nd quartile, Tregs/CD4^+^ T cells 0.0223–0.0296; 3rd quartile, Tregs/CD4^+^ T cells 0.0297–0.0393; 4th quartile, Tregs/CD4^+^ T cells >0.0394; Tregs, regulatory T cells.

Disease relapse occurred in 25 patients with a cumulative incidence at 3 years of 24% (95% CI: 16–33). There was no difference in the cumulative incidence of relapse when analysing either Treg/CD4^+^ T-cell ratios (*P*=0.80) or Treg dose (*P*=0.93) (Figure 3a). Patient diagnosis was associated with relapse (*P*=0.007), with a cumulative incidence of 34% (95% CI: 21–48) and 14% (95% CI: 6–27) for the acute leukaemia and other diagnoses, respectively. Diagnosis was the only independent predictor of relapse (HR, 3.54 (95% CI: 1.49–8.33); *P*=0.004) (Table 4).

The 3-year cumulative incidence of NRM was 24% (95% CI: 16–33), with a median of 154 days (range, 10–747). GvHD was most frequent (45%), followed by infection (27%) (Table 5). Patients transplanted with PBSC grafts containing Treg/CD4^+^ T-cell ratios below the median had a significantly higher NRM (*P*=0.02), with a 3-year cumulative incidence of 35% (95% CI: 21–49) compared with 13% (95% CI: 5–24) in the remainder (Figure 3b). This remained significant after adjusting for differences between the groups (HR, 3.32 (95% CI: 1.18–9.35); *P*=0.03). The observed increased incidence of NRM in patients transplanted with low Treg/CD4^+^ T-cell ratios in the graft was mainly because of increased late NRM (>100 days) with a cumulative incidence at 3 years of 24% (95% CI: 13–37) and 4% (95% CI: 1–13), respectively (*P*=0.006) (Supplementary Table S2). In comparison, the cumulative incidence of early NRM (<100 days) did not differ between the low and high Treg/CD4^+^ T-cell groups, at 11% (95% CI: 2–22) and 9% (95% CI: 3–19), respectively (*P*=0.73). Furthermore, the increased incidence of NRM in patients transplanted with low Treg/CD4^+^ T-cell ratios in the graft was principally because of increased mortality from GvHD. The 3-year cumulative incidence of mortality because of GVHD was 20% (95% CI: 10–32) and 2% (95% CI: 0–10), respectively (*P*=0.007). In multivariate analysis, Treg/CD4^+^ T-cell ratio (HR, 0.30 (95% CI: 0.11–0.85); *P*=0.02) and recipient CMV serology (HR, 3.41 (95% CI: 1.32–8.79); *P*=0.01) remained independent predictors of NRM (Table 4).

### Overall survival

The estimated overall survival at 3 years was 62% (95% CI: 53–72). Thirty-seven patients died at a median of 198 days (range, 10–1484). Patients transplanted with grafts containing Treg/CD4^+^ T-cell ratios above the median had significantly better survival (*P*=0.02), with 3-year overall survival of 75% (95% CI: 63–88) compared with 49% (95% CI: 37–66) in the low Treg/CD4^+^ T-cell cohort (Figure 3c). This remained significant after adjusting for differences between groups (HR 0.48 (95% CI: 0.23–0.97); *P*=0.04). As with NRM, absolute Treg dose in the graft, was not associated with overall survival (*P*=0.25). In multivariate analysis, high Treg/CD4^+^ T-cell ratios in the graft (HR, 0.45 (95% CI: 0.23–0.93); *P*=0.03), younger recipient age (HR, 0.47 (95% CI: 0.23–0.98); *P*=0.04) and negative recipient CMV serology (HR, 0.45 (95% CI: 0.23–0.88); *P*=0.02) were associated with improved overall survival (Table 4).

To demonstrate further the relationship between Tregs in the graft and survival, the study cohort was divided into quartiles using the Treg/CD4^+^ T-cell ratios (Figure 3d and Supplementary Table S3). Overall survival at 3 years was 42% (95% CI: 26–67), 57% (95% CI: 40–81), 67% (95% CI: 50–89) and 83% (95% CI: 69–100) for the first, second, third and fourth quartiles, respectively (*P*=0.03). In multivariate analysis using the lowest quartile as the reference, patients transplanted with grafts containing the highest Treg/CD4^+^ T-cell ratios had significantly improved overall survival (HR, 0.22 (95% CI: 0.06–0.73); *P*=0.01).

### Subgroup analysis

Owing to the potential confounding influence of donor type and alemtuzumab, subgroup analysis was performed for the T-replete (no alemtuzumab) and T-deplete (alemtuzumab) transplants (Table 3). The association between Treg/CD4^+^ T-cell ratios in the graft above the median with improved neutrophil (*P*=0.02), platelet (*P*=0.02) and lymphocyte (*P*=0.004) recovery remained significant in the T-deplete transplants. Conversely, an NS trend (*P*=0.13) towards a lower incidence of acute GvHD (II–IV) with high Treg/CD4^+^ T-cell ratios was observed in T-replete transplants only. CMV reactivation and disease relapse were not significantly associated with the Treg/CD4^+^ T-cell ratio in the graft in either subgroup. The association between high Treg/CD4^+^ T-cell ratios in the graft with improved NRM and overall survival remained in the same direction for both the T-replete and T-deplete transplants, although not reaching statistical significance in these smaller subgroups. The 3-year overall survival in the alemtuzumab-conditioned transplants was 48.6% (95% CI: 34.5–68.3) and 78.3% (95% CI: 63.1–97.1) (*P*=0.07) (HR, 0.44 (95% CI: 0.17–1.10), *P*=0.08) in the low and high Treg/CD4^+^ T-cell groups, respectively (Supplementary Figure S1). Similarly, in the smaller T-replete cohort, the estimated 3-year overall survival was 50.0% (95% CI: 28.4–88.0) and 70.8% (95% CI: 54.8–91.6) (*P*=0.20) (HR 0.50 (95% CI: 0.17–1.49), *P*=0.21), respectively.

## Discussion

Our study of Tregs in PBSC grafts in allogeneic HSCT demonstrates that patients receiving higher proportions of Tregs have substantially better outcomes. Specifically, patients receiving grafts with Treg/CD4^+^ T-cell ratios above the median had a 3-year overall survival of 75% compared with only 49% in the remainder (*P*=0.02). This profound difference in survival was because of a reduction in NRM, with a 3-year NRM of 13% and 35%, respectively (*P*=0.02). Our data showing that successive quartiles of increasing Treg/CD4^+^ T-cell ratios in the graft are associated with progressively better overall survival is consistent with a causal relationship. Furthermore, although not powered for formal subgroup analysis, our data suggest that the association between Treg/CD4^+^ T-cell ratios in the graft with NRM or survival may be observed both T-replete and T-deplete transplants.

What accounts for the improved NRM in patients receiving higher proportions of Tregs? Although less infective-related deaths were observed in patients receiving higher Treg/CD4^+^ T-cell ratios, larger studies will be required to determine whether this is related to improved neutrophil and/or lymphocyte recovery and/or accounts for lower NRM. Nevertheless, the potential mechanisms accounting for the association between graft Treg/CD4^+^ T-cell ratios and haematological recovery are intriguing. In mice, recipient Tregs residing in the bone marrow form a protective niche for transplanted allogeneic HSC, preventing HvG responses.^24^ Moreover, donor Tregs may also facilitate donor haematopoiesis and promote faster lymphocyte recovery by increasing HSC proliferation and cell cycling, possibly through the inhibition of conventional CD4^+^ T cells.^25^ Although, to our knowledge, no direct evidence for such an effect has been published in humans, our observational data suggest that Tregs may influence stem cell growth and development after HSCT. High proportions of Tregs in the graft may also aid immune tolerance, improving immune reconstitution during thymic-independent homeostatic expansion of mature donor T cells and/or thymic-dependent T-cell production.^26, 27, 28^ In our study, there was a trend towards less aGvHD and fewer deaths because of GvHD (1 vs 9) in patients receiving grafts with higher Treg/CD4^+^ T-cell ratios, suggesting that that lower incidence of NRM in this group is due, at least in part, to reduced GvHD-related mortality.

Few clinical studies have examined the effect Tregs in allogeneic HSC grafts on transplant outcomes, although, the published evidence is consistent with our findings. Pastore *et al.*^29^ demonstrated, in 65 allogeneic HSC transplants, that a CD3/Treg ratio in the graft >36 was associated with an increased aGvHD. In an extended study (*n*=74), the cohort with graft CD3/Treg ratios <36 had improved 3-year overall survival (65% vs 31% *P*=0.001) and lower NRM (5% vs 75% *P*<0.0001).^30^ There were 10 deaths from GvHD in the high CD3/Treg group, compared with just one in the low cohort. Other studies suggest a link between absolute Treg counts in allogeneic PBSC grafts and outcome. Wolf *et al.*^31^ showed an association between low Treg doses in the graft and aGvHD in 58 patients. Similarly, Pabst *et al.*^32^ showed that grafts with less than median Tregs had more aGvHD.^32^ These studies are, therefore, broadly consistent with our findings, although we suggest that the ratio of Tregs to other cells (CD3^+^ or CD4^+^ T cells) in the graft is more likely to show an association with clinical outcomes than Tregs alone. Our data showing the association of Tregs/CD4^+^ T cells, but not Treg numbers, with outcomes supports this hypothesis. Furthermore, our findings are consistent with seminal work of Edinger *et al.*^8^ using adoptive transfer of T-cell subsets in mouse models of HSCT to show high Treg/CD4^+^ T-cell ratios permit a potent GvL response while reducing the clinical impact of aGvHD.^8^

The main limitation of our study is the size and heterogeneity of the study cohort and the confounding influence of donor type and alemtuzumab. This detailed phenotypic characterisation of donor grafts was only feasible at one centre, thus restricting the number of patients and dictating the population. However, a larger study would allow clarification of which donor factors influence Tregs in PBSC grafts. Interestingly, in this study, unrelated donors were associated with lower Treg/CD4^+^ T-cell ratios in the graft. Although the explanation remains unclear, it is speculated that the increased time between harvesting and flow cytometry analysis for the unrelated donor grafts may be associated with preferential loss of Tregs and/or downregulation of Treg phenotypic markers, for example, CD25 and/or FOXP3. A larger study would also address the question of whether the proportion of Tregs in the graft has an association with transplant outcomes across different clinical settings. In particular, it would allow predetermined subgroup analysis to confirm whether the influence of graft Tregs is significant in both T-deplete and T-replete transplants. Given the heterogeneity of our study cohort for diagnosis and conditioning regimen, the influence of donor Tregs on disease relapse also remains uncertain. A larger study may clarify whether higher proportions of donor Tregs in the graft reduce NRM, without adversely impacting on relapse, across different disease settings. Use of methylation-specific quantitative PCR to identify demethylated CpG loci within *FOXP3*, specific to Tregs, may simplify quantification of putative Tregs in these larger studies, while excluding transiently activated FOXP3^+^ T cells.^33^

If confirmed by future studies, our observations that higher proportions of Tregs in donor PBSC grafts is associated with improved outcomes could have profound implications for HSCT. It may be speculated that selection of donors with higher peripheral blood Treg levels may provide PBSC grafts with higher proportions of Tregs. Alternatively, additional *ex vivo* isolation of Tregs could be used to increase the proportion of Tregs in the final HSC product. In keeping with this, Martelli *et al.*^20^ reported a study of 43 patients receiving a haploidentical HSCT for acute leukaemia with coinfusion of *ex vivo* isolated Tregs. In the absence of post-transplant immunosuppression, adoptive immunotherapy with Tregs, produced high levels of engraftment and low incidence of acute GvHD and/or relapse compared with historical controls. Adoptive Tregs were also associated with improved lymphoid reconstitution and immunity to opportunistic infections. Whether these approaches improve survival compared with conventional methods and/or in the setting of HLA-matched transplants remains to be established.

In summary, our work shows a robust association between the proportion of Tregs in PBSC grafts and overall survival in allogeneic HSCT. These findings are supported by several other smaller studies. Large-scale, prospective studies of graft composition and outcome are imperative to determine the relationship between Tregs/CD4^+^ T cells in the grafts with clinical outcomes in different allogeneic HSCT settings. If high proportions of Tregs really are causally associated with lower NRM, it may be possible to manipulate Treg/CD4^+^ T-cell ratios in the graft or recipient to substantially improve survival.

## Figures and Tables

**Table 1:** Patient, transplant and graft characteristics

	*Variable*	n
*Recipient*		
Age (years)	Median (range)	48 (18–74)
Gender	Male/female	58/36
CMV serology	Negative/positive	48/46
ABO	O/A/B/AB	40/37/8/9
Disease	Acute leukaemia (AML/ALL)	47 (34/13)
	Other (lymp/Mye/MDS)	47 (27/10/10)
Disease status	Early/other	46/48
		
*Donor*		
Age (years)	Median (range)	40 (19–72)
Gender	Male/female	64/30
CMV serology	Negative/positive	64/30
ABO	O/A/B/AB	41/45/4/4
		
*Recipient/donor*		
Gender (R:D)	Male:female/other	17/77
CMV matching (R:D)	−/−, −/+, +/−, +/+	38/10/26/20
ABO matching	Matched/minor/major/bidirectional	58/18/13/5
HLA matching	Matched/mM HvG/mM GvH/mM Bi	77/2/3/12
		
*Transplant*		
Donor	Sibling/unrelated	38/56
Conditioning	Myeloablative (CyTBI/BuCy)	14 (12/2)
	Reduced intensity (FluMel/FluCy)	80 (79/1)
Graft	PBSC/BM	94/0
Alemtuzumab	No/yes	36/58
		
*PBSC graft*		*× 10^6^/kg*
TNC	Median (range)	1006 (266–3762)
CD34^+^	Median (range)	6.3 (1.1–19.8)
CD3^+^	Median (range)	280 (87–801)
CD3^+^CD4^+^	Median (range)	171 (50–694)
CD3^+^CD8^+^	Median (range)	82 (26–248)
CD19^+^	Median (range)	62 (13–245)
CD3^−^CD56^+^	Median (range)	35 (8–101)
Tregs	Median (range)	4.7 (0.8–20.6)
Tregs/CD4^+^ T cells	Median (range)	0.0296 (0.008–0.086)

Abbreviations: Bi=bidirectional; BuCy=busulphan/cyclophosphamide; CyTBI=cyclophosphamide/TBI; early=CR1, 1st chronic phase or untreated; FluCy=fludarabine/cyclophosphamide; FluMel=fudarabine/melphalan; GvH=graft-versus-host; HvG=host-versus-graft; lymp=lymphoproliferative; mM=mismatched; Mye=myeloproliferative; R:D=recipient:donor; TNC=total nucleated cells; Tregs=regulatory T cells.

**Table 2:** Baseline characteristics of the two study groups

*Transplant variables*	*Low Tregs/CD4*^*+*^ *T cells (*n=*47)*	*High Tregs/CD4*^*+*^ *T* cells (n=*47*)	P*-value*
Treg/CD4^+^ T cells, median (range)	0.022 (0.008–0.029)	0.039 (0.030–0.086)	**<0.0001**
			
*Recipient*			
Age (years), median (range)	49 (18–66)	48 (19–74)	0.73
CMV serology, neg/pos	24/23	24/23	1.00
Disease, acute leuk/other	23/24	24/23	0.84
Disease status, early/other	23/24	23/24	1.00
			
*Donor*			
Age (years), median (range)	39 (19–59)	42 (19–72)	**0.02**
CMV serology, neg/pos	31/16	33/14	0.66
			
*Recipient/donor*			
Gender (R:D), M:F/other	37/10	40/7	0.42
ABO mismatch, minor/major	38/9	38/9	1.00
HLA mismatch (HvG), no/yes	39/8	41/6	0.56
HLA mismatch (GvH), no/yes	38/9	41/6	0.40
			
*Transplant*			
Donor, sibling/unrelated	12/35	26/21	**0.003**
Conditioning, MAC/RIC	9/38	5/42	0.25
Alemtuzumab, no/yes	12/35	24/23	**0.01**
			
*PBSC graft*			
TNC (× 10^8^/kg), median (range)	9.0 (4.0–24.9)	10.7 (2.7–37.6)	0.39
CD34^+^ (× 10^6^/kg), median (range)	6.5 (1.1–9.4)	6.1 (1.5–19.8)	0.99
CD3^+^ (× 10^6^/kg), median (range)	296 (92–793)	253 (87–801)	0.30
CD3^+^CD4^+^ (× 10^6^/kg), median (range)	175 (50–694)	166 (50–504)	0.23
CD3^+^CD8^+^ (× 10^6^/kg), median (range)	85 (36–187)	78 (26–248)	0.32
CD19^+^ (× 10^6^/kg), median (range)	61 (13–245)	63 (20–234)	0.36
CD3^−^CD56^+^ (× 10^6^/kg), median (range)	35 (9–101)	35 (8–100)	0.95

Abbreviations: acute leuk=acute leukaemia; HvG=host-versus-graft; M:F=male:female; MAC=myeloablative; R:D=recipient:donor; RIC=reduced intensity; TNC=total nucleated cells.

Low Tregs/CD4^+^ T cells (<0.0296); High Tregs/CD4^+^ T cells (>0.0296).

Significant variables (*P*<0.05) shown in bold.

**Table 3:** Univariate analysis

*Outcome*	*Tregs/CD4+ T cells*	*All patients (*n=*94)*			*T-replete (*n=*36)*			*T-deplete (*n=*58)*		
		*%*	*95% CI*	P-*value*	*%*	*95% CI*	P-*value*	*%*	*95% CI*	P-*value*
Neutrophil recovery (day 30)	<0.0296	87	73–94		92	34–99		86	68–94	
				**0.046**			0.33			**0.02**
	>0.0296	98	64–100		96	34–100		100	100–100	
										
Platelet recovery (day 60)	<0.0296	87	73–94		100	100–100		83	65–92	
				**0.03**			0.45			**0.02**
	>0.0296	98	67–100		96	43–100		100	100–100	
										
Lymphocyte recovery (day 180)	<0.0296	61	45–73		92	33–99		59	31–64	
				**0.001**			0.64			**0.004**
	>0.0296	83	68–92		92	64–98		70	45–85	
										
CMV activation (1 year)	<0.0296	45	30–59		42	14–68		46	29–61	
				0.89			0.99			0.74
	>0.0296	45	30–58		42	22–61		48	26–67	
										
Acute GvHD (day 100)	<0.0296	38	25–52		42	14–68		37	21–53	
				0.11			0.13			0.51
	>0.0296	23	13–36		17	5.0–34		30	13–50	
										
Chronic GvHD^a^ (3 years)	<0.0296	74	56–85		80	36–96		71	50–85	
				0.79			0.51			0.79
	>0.0296	74	58–85		77	52–90		71	45–87	
										
Relapse (3 years)	<0.0296	25	13–38		17	2–43		28	14–44	
				0.80			0.70			0.83
	>0.0296	23	12–36		21	7–39		26	10–35	
										
Non-relapse mortality (3 years)	<0.0296	35	21–49		33	9–60		35	20–51	
				**0.02**			0.16			0.07
	>0.0296	13	5–24		13	3–29		13	3–30	
										
Overall survival (3 years)	<0.0296	49	37–66		50	28–88		49	35–68	
				**0.02**			0.20			0.07
	>0.0296	75	63–88		71	55–92		78	63–97	

Abbreviations: CI=confidence interval; Tregs=regulatory T cells.

Low Tregs/CD4^+^ T cells (<0.0296); high Tregs/CD4^+^ T cells (>0.0296).

a Only patients with survival >100 days (*n*=83).

Significant variables (*P*<0.05) shown in bold.

**Table 4:** Multivariate analysis

*Outcome*	*Variable*		*HR*	*95% CI*	P*-value*
Neutrophil recovery^a^	Tregs/CD4^+^ T cells	>Median	0.52	0.37–0.79	**0.002**
	CD34^+^ dose	>Median	0.65	0.44–0.96	**0.03**
					
Platelet recovery^b^	Tregs/CD4^+^ T cells	>Median	0.51	0.34–0.76	**<0.001**
	CD34^+^ dose	>Median	0.60	0.40–0.88	**0.01**
	CD56^+^ dose	>Median	1.52	1.01–2.27	**0.046**
					
Lymphocyte recovery^c^	Tregs/CD4^+^ T cells	>Median	0.54	0.34–0.86	**0.009**
	Donor	Sibling	0.30	0.16–0.55	**<0.001**
	Donor gender	Female	0.59	0.38–0.93	**0.02**
					
CMV activation^d^	Tregs/CD4^+^ T cells	>Median	1.36	0.73–2.54	0.33
	Recipient CMV	Seropositive	21.5	6.66–69.9	**<0.001**
	Donor CMV	Seropositive	2.50	1.43–4.36	**0.001**
					
Acute GvHD^e^	Tregs/CD4^+^ T cells	>Median	0.58	0.26–1.31	0.19
	Stage of disease	Other	3.37	1.48–7.66	**0.004**
					
Chronic GvHD^f^	Tregs/CD4^+^ T cells	>Median	0.97	0.59–1.60	0.90
					
Relapse^g^	Tregs/CD4^+^ T cells	>Median	1.31	0.59–2.93	0.51
	Diagnosis	Leukaemia	3.54	1.49–8.33	**0.004**
					
Non-relapse mortality^h^	Tregs/CD4^+^ T cells	>Median	0.30	0.11–0.85	**0.02**
	Recipient CMV	Seropositive	3.41	1.32–8.79	**0.01**
					
Overall survival^i^	Tregs/CD4^+^ T cells	>Median	0.45	0.23–0.93	**0.03**
	Recipient age	>Median	2.13	1.02–4.35	**0.04**
	Recipient CMV	Seropositive	2.22	1.14–4.35	**0.02**

Abbreviations: CI=confidence interval; HR=hazard ratio; Tregs=regulatory T cells.

Variables included in the multivariate models were the proportion of Tregs (Tregs/CD4^+^ T cells) in the grafts (adjusted for donor age and donor type (sibling/unrelated); adjustment for alemtuzumab could not be included as donor type and alemtuzumab are confounded) and

a CD34^+^ cell dose;

b CD34^+^ cell dose, CD3^−^CD56^+^ cell dose;

c Donor gender, Treg dose;

d Recipient CMV serology, donor CMV serology, HLA-mismatch in HvG direction and HLA-mismatch in GvH direction;

e Stage of disease at transplant;

f None;

g Diagnosis;

h Recipient CMV serology and HLA-mismatch in HvG direction;

i Recipient age, recipient CMV serology and HLA-mismatch in HvG direction.

Significant variables (*P*<0.05) shown in bold.

**Table 5:** Cause of mortality

*Cause*	*All patients* (n=*94)*		*Low Tregs/CD4*^*+*^ *T* cells (n=*47)*		*High Tregs/CD4*^*+*^ *T* (n=*47)*	
	n	*%*	n	*%*	n	*%*
All mortality	37	100	24	100	13	100
Relapse	15	41	8	33	7	54
Non-relapse	22	59	16	66	6	46
GvHD	10	27 (45)	9	38 (56)	1	8 (17)
Infection	6	16 (27)	4	17 (25)	2	15 (33)
Other	6	16 (27)	3	13 (18)	3	23 (50)

Abbreviations: Low Tregs/CD4^+^=below the median (<0.0296); High Tregs/CD4^+^ T=above the median (>0.0296).

Numbers within parentheses are the percentage of non-relapse mortality only.
