# Supplementary material for: High proportions of regulatory T cells in PBSC grafts predict improved survival after allogeneic haematopoietic SCT
Source: Bone Marrow Transplant. 2015 Sep 21;51(1):110–8. doi: 10.1038/bmt.2015.215 (PMC4705424; doi:10.1038/bmt.2015.215)
Supplement: Supplementary Information [file bmt2015215x1.doc]

**Supplementary Material**

**Methods**

*Transplant details*

Eighty (85%) transplants were performed using a reduced-intensity conditioning (RIC) regimen containing Fludarabine 125 mg/m^2^ and Melphalan 140 mg/m^2^; or Fludarabine 120 mg/m^2^ and Cyclophosphamide 1200 mg/m^2^. Fourteen (15%) used myeloablative conditioning (MAC) with Cyclophosphamide 120 mg/kg and 14.4 Gy TBI; or Cyclophosphamide 120 mg/kg and Busulphan 16 mg/kg. Patients with MAC received Methotrexate 15 mg/m^2^ on day +1 and 10 mg/m^2^ on day +3, +6, and +11, whilst Fludarabine/Melphalan sibling allografts received Methotrexate 5 mg/m^2^ on day +1, +3, +6, and +11.

*Flow cytometry*

PBSC grafts were analysed by flow cytometry using CD45/CD3/CD4/CD8 Multitest with Trucount tubes (342447) and anti-CD3 PE-Cy7 (557851), CD4 FITC (555346), CD19 PE (555413), CD56 APC (555518) [BD Biosciences, Oxford, UK], and CD8 APC Alexa-Fluor 750 (27-0088, eBiosciences, Hatfield, UK). To quantify Tregs, defined phenotypically as CD3^+^CD4^+^CD8^−^CD25^+^FOXP3^+^CD127^dim/−^ cells, peripheral blood mononuclear cells (PBMC) were isolated from PBSC grafts using Ficoll-Paque and stained using anti-CD3 PE-Cy7 (557851), CD4 FITC (555346), CD25 PE (557741) [BD Biosciences]; CD8 APC-Alexa-Fluor 750 (27-0088), and CD127 PerCP-Cy5.5 (45-1278) [eBiosciences]. Stained PBMC were washed, fixed and permeabilized, and stained with anti-FOXP3 APC (130-093-013, Miltenyi Biotech, Bisley, UK). Flow cytometry was performed on an LSR II Flow Cytometer using FACS Diva software (BD Biosciences). Staining controls were fluorescence-minus-one, isotype controls, and antigen negative cells.

*Statistics*

Sample size was calculated with overall survival as the primary outcome. The historical 3-year overall survival for allogeneic HSCT for Oxford University Hospitals NHS Trust is 60-65%. Based upon these data, this study was designed to detect a difference of 25%, with an estimated 3-year overall survival of 50% and 75% in the low and high Treg/CD4^+^ T-cell groups, respectively. With a one-sided model using an α value of 0.05 and β value of 0.2, the estimated sample size was 45 in each arm.

Variables initially considered in univariate analysis were recipient and donor age; sex mismatch (female donor to male recipient vs other); ABO mismatch (matched/minor vs major/bidirectional); recipient and donor CMV serology; disease (acute leukaemia vs other); disease stage (Early (CR1/chronic phase/untreated) vs Other (≥ CR2/partial response/active disease); conditioning (RIC vs MAC); donor (sibling vs unrelated). Graft variables considered were CD34^+^, CD3^+^, CD19^+^, CD3^−^CD56^+^, Treg (CD3^+^CD4^+^CD25^+^FOXP3^+^CD127^dim/−^) dose, and the Treg/CD4^+^ T-cell ratio.

**Table S1.** Analysis of the PBSC graft contents.

| **A. Cell type** | **Treg dose** | | **Treg/CD4^+^ T-cell ratio** | |
| --- | --- | --- | --- | --- |
|  | ***r*** | ***P*** | ***r*** | ***P*** |
| TNC | 0.45 | **< 0.0001** | 0.03 | 0.78 |
| CD34^+^ | 0.13 | 0.22 | 0.02 | 0.86 |
| CD3^+^ | 0.65 | **< 0.0001** | −0.10 | 0.35 |
| CD19^+^ | 0.53 | **< 0.0001** | 0.12 | 0.25 |
| CD3^−^CD56^+^ | 0.50 | **< 0.0001** | 0.12 | 0.25 |

| **B. Univariate** | **Variable** | | **Median** | **Range** | ***P*** |
| --- | --- | --- | --- | --- | --- |
| Treg Counts (×10^8^) | Donor | Sibling | 4.35 | 1.73-13.78 | **0.01** |
|  |  | Unrelated | 3.48 | 0.48-10.54 |  |
|  | Harvest | One day | 3.51 | 0.48-9.62 | **< 0.001** |
|  |  | Two days | 6.76 | 2.36-13.87 |  |
| Treg/CD4^+^ T cells | Donor | Sibling | 0.033 | 0.011-0.086 | **0.04** |
|  |  | Unrelated | 0.026 | 0.008-0.061 |  |
|  | Gender | Male | 0.030 | 0.008-0.086 | 0.07 |
|  |  | Female | 0.028 | 0.014-0.045 |  |

| **C. Multivariate** | **Variable** | **B** | **SE B** | **β** | ***P*** | ***R*^2^** |
| --- | --- | --- | --- | --- | --- | --- |
| Treg Counts (×10^8^)* | Constant | 0.84 | 0.72 |  | 0.24 | 0.24 |
|  | Harvest | 3.02 | 0.56 | 0.49 | **< 0.001** |  |
| Treg/CD4^+^ T cells^#^ | Constant | −1.44 | 0.04 |  |  | 0.09 |
|  | Donor | −0.10 | 0.04 | −0.28 | **0.01** |  |
|  | Gender | −0.10 | 0.04 | −0.24 | **0.03** |  |

(A) Spearman correlation (*r*) between Treg dose (× 10^6^/kg) or proportion of Tregs (Tregs/CD4^+^ T cells), and the dose (× 10^6^/kg) of the main cell populations in the graft. (B) Univariate analysis of the Treg counts (× 10^8^) and proportion of Tregs (Tregs/CD4^+^ T cells) in the graft. Analysed by Mann-Whitney test. (B) Multivariate linear regression of Treg counts (× 10^8^) and proportion of Tregs (Tregs/CD4^+^ T cells)) in the graft. *Data were transformed using a √ transformation; ^#^Data were transformed using a log10 [x/(1−x)] transformation; Tregs, regulatory T cells.

**Table S2. Timing and cause of mortality.**

| **Low Treg/CD4^+^ T-cell ratio** | | **High Treg/CD4^+^ T-cell ratio** | |
| --- | --- | --- | --- |
| *Day* | *Cause* | *Day* | *Cause* |
| 10 | Infection | 18 | Venoocclusive disease |
| 16 | Infection | 20 | Infection |
| 35 | GvHD | 25 | Infection |
| 43 | GvHD | 59 | Pneumonitis |
| 44 | Relapse | 146 | Relapse |
| 74 | GvHD | 157 | Relapse |
| 116 | Relapse | 171 | Relapse |
| 143 | Relapse | 199 | Relapse |
| 145 | Thrombosis | 241 | GvHD |
| 161 | Infection | 304 | Relapse |
| 163 | GvHD | 339 | PTLD |
| 198 | Relapse | 427 | Relapse |
| 221 | Relapse | 1484 | Relapse |
| 226 | GvHD |  |  |
| 240 | Neuropathy |  |  |
| 245 | GvHD |  |  |
| 252 | Infection |  |  |
| 256 | GvHD |  |  |
| 258 | Relapse |  |  |
| 315 | GvHD |  |  |
| 317 | GvHD |  |  |
| 566 | Relapse |  |  |
| 747 | Intracranial haemorrhage |  |  |
| 845 | Relapse |  |  |

Tregs, regulatory T cells; PTLD, post-transplant lymphoproliferative disorder.

**Table S3.** Overall survival according to the proportion of Tregs (Tregs/CD4^+^ T cells) in the PBSC grafts (quartiles).

| **A. Univariate analysis** | | | |
| --- | --- | --- | --- |
| **Group** | **Tregs/CD4^+^ T cells** | **3-year OS (%)** | **95% CI** |
| 1^st^ quartile | 0.0081-0.0222 | 42 | 26-67 |
| 2^nd^ quartile | 0.0223-0.0295 | 57 | 40-81 |
| 3^rd^ quartile | 0.0296-0.0393 | 67 | 50-89 |
| 4^th^ quartile | 0.0394-0.0856 | 83 | 69-100 |

| **B. Multivariate analysis** | |  |  |  |
| --- | --- | --- | --- | --- |
| **Variable** | | **HR** | **95% CI** | ***P*** |
| **Tregs/CD4^+^ T cells** | 1st quartile | 1.00 |  |  |
|  | 2nd quartile | 0.74 | 0.30-1.82 | 0.51 |
|  | 3rd quartile | 0.63 | 0.25-1.56 | 0.32 |
|  | 4th quartile | 0.22 | 0.06-0.73 | **0.01** |
| **Recipient CMV** | Seronegative | 1.00 |  |  |
|  | Seropositive | 2.23 | 1.07-4.66 | **0.03** |

(A) Kaplan-Meier estimation of 3-year overall survival according to the proportion of Tregs (Tregs/CD4^+^ T cells) in the grafts, divided by quartiles. (B) Multivariate analysis of overall survival. Variables included in the initial model were the Treg/CD4^+^ T-cell ratios in the peripheral blood stem cell grafts (quartiles) adjusting for significant differences between the groups (donor age, donor gender, and CD3^−^CD56^+^ cell dose) and variables with *P* < 0.10 in univariate analysis (recipient age, recipient CMV serology, and HLA-mismatch in HvG direction). Tregs, regulatory T cells; OS, overall survival.

**A. No Alemtuzumab (*n*=36) B. Alemtuzumab (*n*=58)**

***P* = 0.20**

***P* = 0.07**

**Figure S1.** Overall survival in T-replete and T-deplete transplants according to the proportion of Tregs (Tregs/CD4^+^ T cells) in the graft. (A) No Alemtuzumab conditioning; (B) Alemtuzumab conditioning. Low %Tregs, Tregs/CD4^+^ T cells below the median (dotted line); High %Tregs, Tregs/CD4^+^ T cells above the median (solid line); Tregs, regulatory T cells.
